# A Constitutive Model of Plate-Like Entangled Metallic Wire Material in Wide Temperature Range

**DOI:** 10.3390/ma12162538

**Published:** 2019-08-09

**Authors:** Zheyu Ding, Hongbai Bai, Yiwan Wu, Zhiying Ren, Yichuan Shao

**Affiliations:** Engineering Research Center for Metal Rubber, School of Mechanical Engineering and Automation, Fuzhou University, Fuzhou 350116, China

**Keywords:** entangled metallic wire material, Johnson–Cook, Sherwood–Frost, quasi-static compression, high temperature

## Abstract

Entangled metallic wire material (EMWM) is a kind of porous damping material. To promote the engineering application of EMWM, it is necessary to establish the constitutive model of EMWM to estimate its mechanical properties. In this paper, a series of quasi-static compression experiments for plate-like EMWM specimens made of austenitic stainless steel wire (06Cr19Ni10) with different densities were carried out in the temperature range of 20–500 °C. It was found that the stiffness of the plate-like EMWM would increase with the increases in the ambient temperature. The non-linear characteristics of the force–displacement curve of the plate-like EMWM would be weakened. Taking the spatial structural characteristics of the plate-like EMWM and the influence of the thermal expansion of the structure into account, a new constitutive model for plate-like EMWM was presented by the combination of the Johnson–Cook model and the Sherwood–Frost constitutive framework model. The accuracy of the model was verified by the experimental data under different temperatures. The results show that the calculated results of the model are consistent with the experimental results. This model can provide an effective theoretical basis for predicting the mechanical properties of plate-like EMWM and guiding its design.

## 1. Introduction

Entangled metallic wire material (EMWM) is a new damping material made of various kinds of metal wire helixes. The EMWM is sometimes referred to as ‘metal rubber (MR)’ [1] or ‘elastic porous wire mesh (EPWM)’ [2]. The EMWM has the characteristics of high elasticity and large damping like rubber. It also has excellent physical and mechanical properties like metal. The inner space network structure of the EMWM is similar to that of macromolecular natural rubber. When the EMWM is excited by external forces, the wires will slide, fractionate and extrude, and the vibration energy will be dissipated and converted into frictional heat energy. Compared with natural rubber, EMWM has better high–low temperature adaptability [2]. The comprehensive properties enable this material to be used in extremely rough environments, such as the vibration control of thin-wall casings for modern aero-engines [3], the vibration control of power turbine rotors for turboprop engines [4], isolation mounting of electronic components in satellites and launch vehicles [5], and the sealing of rotors for turbo-machinery [6].

The mechanical properties of EMWM are complex. For the inner spatial structure of the EMWM, its mechanical properties are non-linear [7]. On the other hand, the EMWM has viscoelastic properties, which are due to the existence of friction and slip between wire helixes. How to accurately describe the constitutive relationship of materials is the key to ensure the reliability of numerical simulation analysis of the mechanical properties of materials. Li [8] constructed the non-linear constitutive equation for elastic porous metal rubber from the theory of porous material, the non-linear theory of dry friction and the curved slender beam model, but the influence of ambient temperature was not taken into account. Under different temperatures, the expansion coefficient of the metal wire helix will be changed. Moreover, the strain of the metal is coupled with the ambient temperature. Therefore, the mechanical model of the EMWM is more complicated with the consideration of ambient temperature.

In the past, the mechanical properties of different materials under different temperatures have been investigated extensively by scholars [2,9,10,11,12,13,14,15,16]. Wang [9] discussed the effects of distribution type of reinforcements, core-to-face sheet thickness ratio, temperature variation, foundation stiffness and in-plane boundary conditions on the nonlinear vibration characteristics of sandwich plates with piece-wise functionally graded graphene-reinforced composite face sheets. Do [10] presented new numerical results of mechanical behavior for functionally graded sandwich plates in high temperature and investigated material combinations and stress distribution of sandwich plates with functionally graded materials faces. Hou [2] proposed a damping characteristic measurement method for EMWM based on the hysteresis curve decomposition, and measured the loss factor in a wide temperature range (−70 to 300 °C). Li [11] investigated the effects of ambient temperature, frequency and amplitude on the compression performance of a kind of knitted-dapped metal rubber samples by static and dynamic compression tests under different temperatures.

The Johnson–Cook model [17] and Sherwood–Frost model [18] have been widely used to describe the stress–strain relationship of metal porous materials. The Johnson–Cook model is an empirical constitutive model. The structure of the Johnson–Cook model is simple and the physical meaning of each term is clear. The Johnson–Cook model was used to describe the strength limit and failure process of metallic materials under large strain, high strain rate and high temperature [19,20,21]. Its expression is as follows:

(1) $$\sigma =\left(A+B\varepsilon^{n}\right)\left(1+C\ln{\dot{\varepsilon }}^{*}\right)\left(1-T^{*m}\right)$$

where A is initial yield stress, B is hardening constant, C is strain rate constant, m is thermal softening exponent, n is hardening exponent, σ is stress, ε is strain, $\dot{\varepsilon }$ is strain rate, and T is temperature, ${\dot{\varepsilon }}^{*}=\dot{\varepsilon }/{\dot{\varepsilon }}_{0}$,$\;{\dot{\varepsilon }}_{0}=1.0\;s^{-1}$,$\;T^{*}=\left(T-T_{room}\right)/\left(T_{melt}-T_{room}\right)$,$\;T_{room}$ is room temperature, $T_{melt}$ is the melting point of the material.

In the 1990s, Sherwood and Frost proposed a more comprehensive framework of the constitutive model for aluminum foam with the consideration of the density and ambient temperature. It was often used in the establishment of constitutive models of metal foams [22,23,24]. The Sherwood–Frost model is as follows:

(2) $$\sigma =H\left(T\right)G\left(\rho \right)M\left(\varepsilon ,\dot{\varepsilon }\right)f\left(\varepsilon \right)$$

where ρ is the density, H(T) is the temperature softening term, G(ρ) is the density term, $M\left(\varepsilon ,\dot{\varepsilon }\right)$ is the strain rate enhancement term, and f(ε) is the shape function.

The mechanical properties of EMWM are similar to that of the metal foams. But, the contact states between the wire helixes inside EMWM is complicated. Therefore, it is unreasonable to apply the relevant empirical formula to EMWM directly. On the other hand, in the previous researches, the ambient temperature is not taken into account in the constitutive models for EMWM.

In this paper, stress–strain curves of three kinds of plate-like EMWM with different densities ($1.905\;g/cm^{3}$,$\;2.222\;g/cm^{3}$,$\;2.540\;g/cm^{3}$) were obtained in a wide temperature range (20–500 °C) through a series of quasi-static compression experiments. A new constitutive model for EMWM would be presented by the combination of the Johnson–Cook constitutive equation and the Sherwood–Frost constitutive model. In addition, the temperature term in the constitutive model would be modified with the consideration of the thermal expansion of the wire helixes inside the EMWM. Finally, the correctness of the constitutive model was validated by comparisons between the predicting data and the experimental data under different temperatures.

## 2. Quasi-Static Test for Plate-like Entangled Metallic Wire Material (EMWM) under Different Temperatures

### 2.1. EMWM Specimens

Austenitic stainless steel wire (06Cr19Ni10) with wire diameter of 0.3 mm was used to manufacture plate-like EMWM specimens via a four-step processes [25]. (1) The ordinary straight metal wire is encircled in a tight helix with the processing principle of the helix spring. (2) The tight helix is tensioned and weaved in a crisscross pattern to obtain a rough porous base material. (3) The rough samples are placed into a specially designed mold and shaped into final form by applying a compressive force to obtain a primary EMWM. (4) The primary EMWM samples are post-processed (ultrasonic clean, heat treatment) to obtain the final EMWM

**Figure 1:**
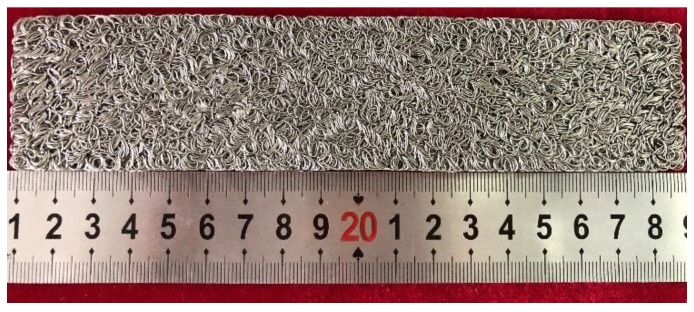
Plate-like EMWM specimen.

The dimensions of the plate-like EMWM specimens are 175 mm × 40 mm × 4.5 mm. The manufacturing parameters of the specimens are shown in Table 1. The plate-like EMWM sample is shown in Figure 1. 

**Figure 2:**
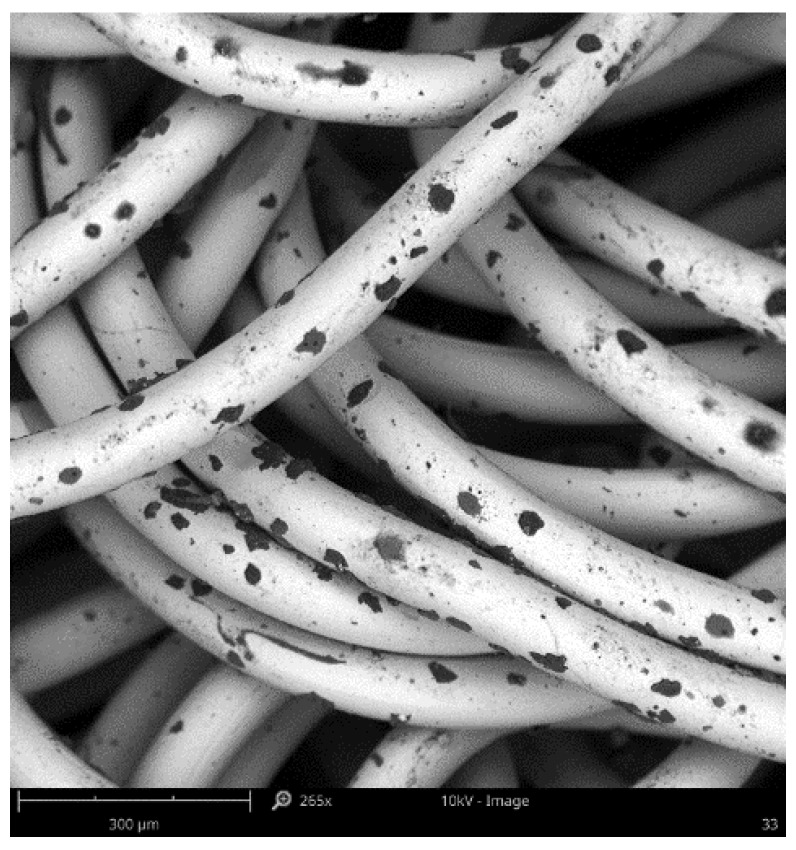
Scanning electron microscope (SEM) image of EMWM.

From the mesoscopic point of view, as shown in Figure 2, it is evident that the EMWM can be divided into multi-segment curved beams in contact points. These curved beams are overlapped and interconnect closely with each other. The mechanical properties of the EMWM are determined by the elastic force of their deformation and interaction force between them [26].

### 2.2. Experiment

#### 2.2.1. Testing Equipment

**Figure 3:**
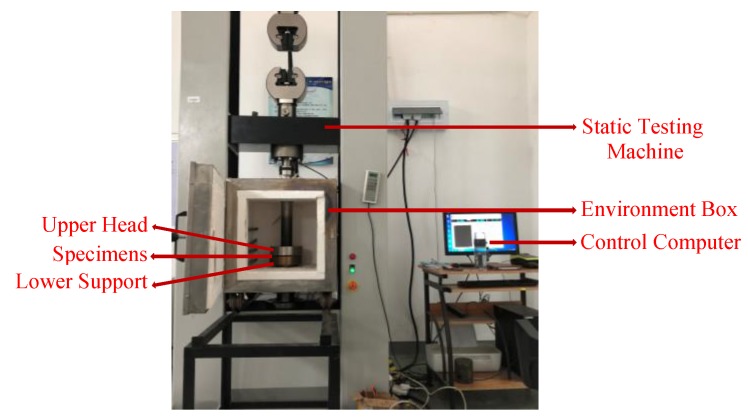
Experiment equipment.

**Figure 4:**
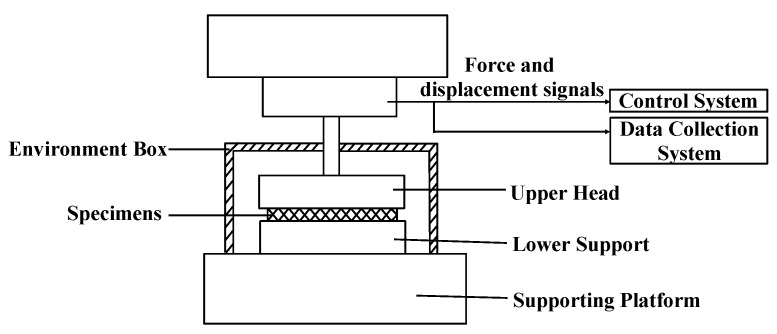
Schematic of static compression testing system.

A series of quasi-static tests for plate-like EMWM specimens under different temperatures were carried out using a WDW-T200 electronic universal testing machine and a high-temperature testing box (Jinan Tianchen Testing Machine Manufacturing Co., Ltd., Jinan, China), as shown in Figure 3 and Figure 4. The maximum compression force is 200 kN, the maximum temperature is 800 °C, the displacement resolution is 0.001 mm, the load resolution is 1 N, and the temperature resolution is 1 °C.

#### 2.2.2. Experimental Methods 

The WDW-T200 was used in the displacement control mode to perform displacement loading on the specimens. During each test, the loading speed was controlled at 1 mm/min. To decrease the influence of uneven contact surface between the EMWM specimen and the test holders (upper head and lower support), each specimen was pre-compressed directly with a small pre-pressure (20 N) for 3 min before the test. Then the sensors reading were reset, and the specimen was loaded and unloaded at a constant speed. To control the test variables, the maximum loading force was set as 30% of the forming pressure of the specimen 3. The value of the pre-pressure (20 N) is far less than the maximum loading value (30 kN), thus the effect of pre-pressure on the test results can be ignored. Each specimen was loaded and unloaded under different ambient temperatures (20 °C, 100 °C, 200 °C, 300 °C, 400 °C and 500 °C). After each temperature was kept for 30 minutes, the value of the force sensor, which was caused by thermal expansion, was recorded, and then the force and displacement sensor readings were cleared and the corresponding quasi-static test was started.

During the test, due to the pre-pressure (20 N), the EMWM was constrained by the upper head and lower support of the electronic universal testing machine in the molding direction. The thermal stress of the EMWM changes with the change of the ambient temperature, and then the stress–strain curves (force–displacement curves) will not start from zero strain and zero stress (zero force and zero displacement). To facilitate the analysis, the value of thermal stress was recorded before each test, and then the force–displacement curves start from zero force and zero displacement. It is noted that the thermal stress would be considered in temperature softening terms. 

**Figure 5:**
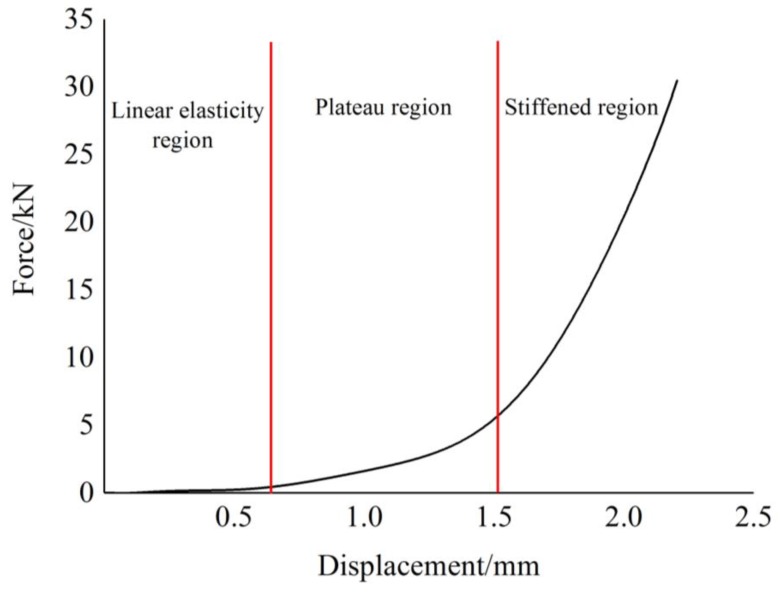
Sketch of the loading process of EMWM.

Figure 5 is the sketch of the loading process of the plate-like EMWM. The loading curve can be divided into three regions: linear elasticity region, plateau region, and stiffened region. When the external load on the EMWM increases from zero, the force grows near linearly, and the EMWM is in the linear region; as the external force continues to increase, contact deformation occurs inside the EMWM, the force grows slowly and non-linear characteristic of the curve become gradually obvious with the increases of the displacement, the EMWM is in the plateau region; the stiffened region appears when the EMWM is subjected to a large load, in this region, the stiffness of the EMWM increase sharply. 

The actual stiffness of the EMWM is the tangent slope of each point on the curve. In order to facilitate the analysis of the change of the stiffness of the EMWM with temperature, the secant stiffness is used to indicate the change of stiffness. 

**Figure 6:**
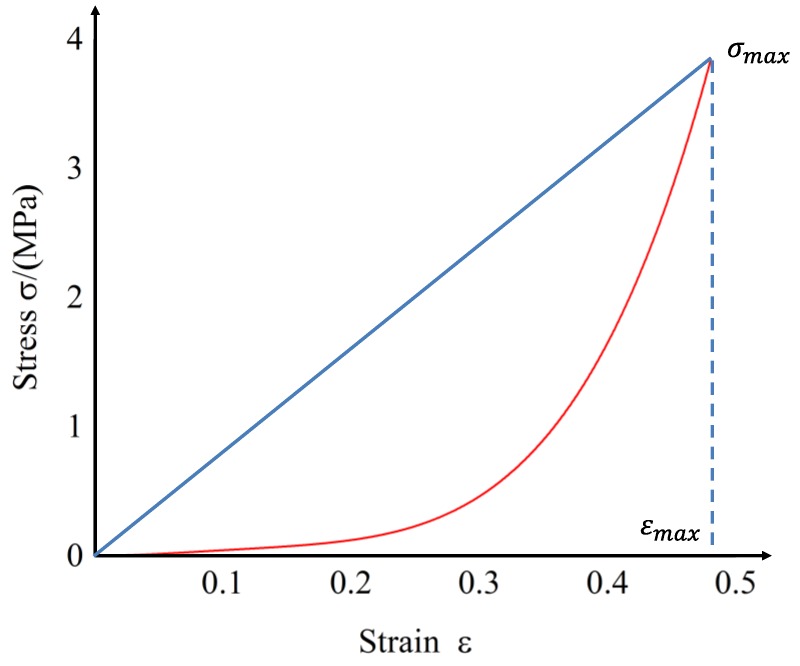
Secant of stress–strain curve.

To simplify the calculation process, the ratio of the difference between the maximum value and the minimum value of the stress–strain curve stress and the difference between the maximum and minimum strain values is used to represent the secant stiffness during the same loading process. Considering that the stiffness change during the loading and unloading process is independent of the initial thermal stress, the influence of thermal stress is ignored in the calculation and the curve starts from the origin of the coordinates. As shown in Figure 6, the secant stiffness can be expressed as

(3) $$\bar{k}=\frac{\sigma_{max}}{\varepsilon_{max}}$$

To facilitate the establishment of the constitutive model for EMWM, the force–displacement curves of the plate-like EMWM can be re-expressed in the form of stress–strain curves. The following conversion equations are used:

(4) $$\sigma =\frac{F}{S}$$

(5) $$\varepsilon =\frac{h-x}{h}$$

where *F* is the loading force, *S* is the cross-sectional area, *h* is the thickness of specimen, *x* is the displacement.

#### 2.2.3. Results and Discussion 

**Figure 7:**
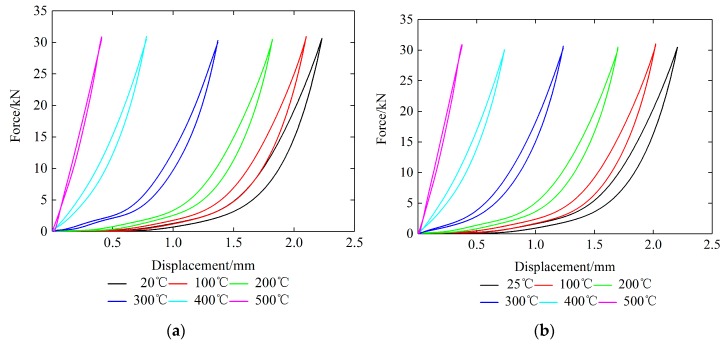

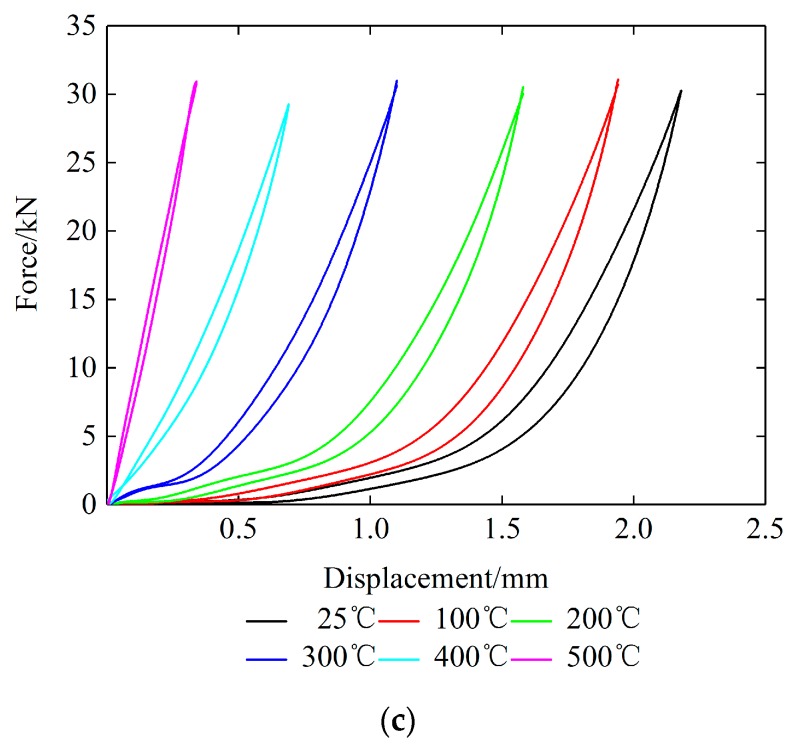
Force–displacement curves of each specimen under different temperatures: (**a**) Specimen 1; (**b**) Specimen 2; (**c**) Specimen 3.

The force–displacement curves of one loading–unloading cycle of each plate-like EMWM specimen under different ambient temperatures are shown in Figure 7.

**Figure 8:**
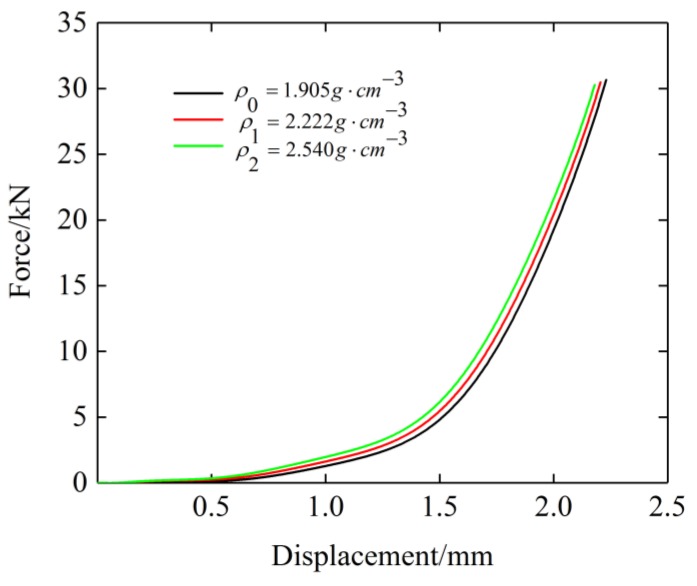
Force–displacement curves of each specimen under the same temperature (20 °C).

Figure 8 shows that under the same ambient temperature and deformation, the greater the density of the specimen, the greater the restoring force would be. As the density of the EMWM increases, the number of contact points of curved beams increases for a given volume. From the mesoscopic point of view, as the number of contact points increases, the effective length of the curved beam decreases. The results obtained by Cao et al. [26] shown that the equivalent stiffness of the curved beam increases with the decreases of the effective length of the curved beam. The EMWM is composed of multi-segment curved beams in contact points, as mentioned above. The stiffness of the EMWM will increase with the increase of the equivalent stiffness of the curved beams.

**Figure 9:**
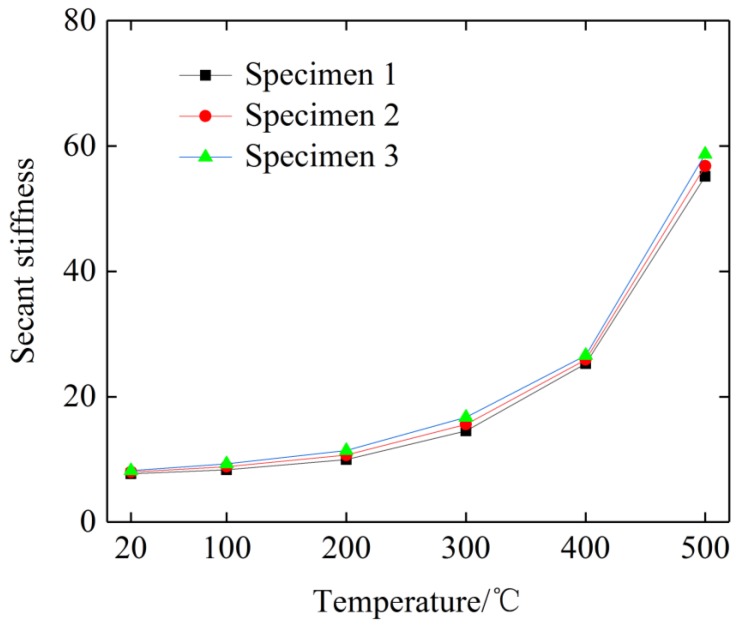
The trend of secant stiffness with temperature.

**Figure 10:**
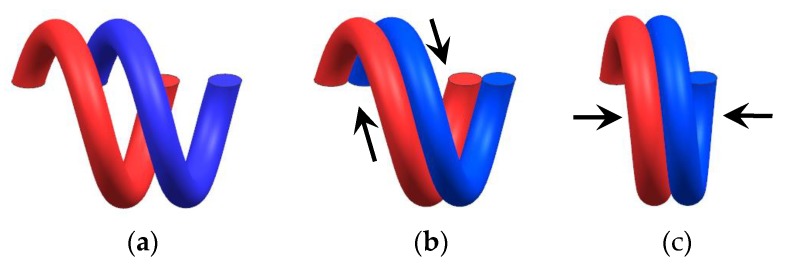
Three types of interaction during compression according to the EMWM micromechanics model: (**a**) non-contact, (**b**) slip contact, and (**c**) stick contact.

Figure 7 and Figure 9 also show that under different ambient temperatures, the restoring force and stiffness of the specimen increases with the increase of temperature. For austenitic stainless steel, the value of the properties decreases with an increase in temperature, but in the temperature range of 300–550 °C this trend is not followed. The ultimate tensile strength is seen to decrease until the temperature of 300 °C and further seen to become approximately constant until 550 °C. The yield strength is also found to decrease drastically and become constant and again decrease. This irregular variation in the properties is due to the effect of dynamic strain ageing [27]. In general, the strength of austenitic stainless steel drops almost 30% by increasing the temperature from 50 °C to 500 °C. But for the EMWM, the friction coefficient of the wire helixes increases with the increase of the ambient temperature from 20 °C to 200 °C. When the temperature is greater than 200 °C, a dense enamel oxidation film will form on the surface of the wire helixes, and then the friction coefficient of the wire helixes will gradually reduce. On the other hand, with the increase of ambient temperature, the amount of thermal expansion of the wire helixes will become larger. As shown in Figure 10, there are three types of interaction (non-contact, slip, and stick) between the wire helixes in the EMWM during compression [28,29]. The effective stiffness for slip and stick is greater than that for non-contact. Thus, as the amount of thermal expansion of the wire helixes increases, the interaction type of the wire helixes will change gradually (from non-contact to slip, from slip contact to stick contact). This means that the non-contact status occupies a smaller percentage and the number of contact points increases. As the ambient temperature increases, the linear elasticity region and the plateau region gradually decreases, and the EMWM is in the stiffened region. The stiffness characteristic of EMWM is gradually similar to solid structures, and the non-linear characteristics of the force–displacement curve of EMWM would be weakened. Due to the influence of the internal properties of the EMWM, the load-bearing capacity of the EMWM increases with the increase of temperature from 50 °C to 500 °C until the degradation in mechanical properties of the base material counters the strengthening effect of the EMWM. Therefore, the load-bearing capacities of the EMWM increase with the increase of the ambient temperature from 50 °C to 500 °C.

## 3. Constitutive Equation of Plate-Like EMWM

The mechanical properties of the material are the result of the combination of strain strengthening effects, strain rate effects, and temperature effects. The Johnson–Cook model has a simple structural form and a clear representation of each physical quantity, but it simplifies the strain enhancement into a high-order non-linear enhancement and does not consider the effect of density. The Sherwood–Frost constitutive model expresses strain strengthening as a shape function in the form of series and proposes a density term whose general function form is shown in Equation (6) [24].

(6) $$G\left(\rho \right)=B(\frac{\rho }{\rho_{0}}{)}^{Y}$$

where B and Y are the correlation coefficients of the density term, ρ is specimen density, $\rho_{0}$ is reference density. 

The Sherwood–Frost constitutive model makes the influence of the deformation and density of the material on the mechanical properties of the porous material can be expressed more accurately. Previous researches have shown that the combination of the Johnson–Cook model and the Sherwood–Frost constitutive model with proper modification can more accurately describe the mechanical properties of porous materials [30,31,32].

To accurately predict the mechanical properties of the plate-like EMWM, the temperature softening term expression in the Johnson-Cook model is incorporated into the Sherwood–Frost constitutive framework model, and $H\left(T\right)=1-T^{*m}$ is assumed. On the other hand, the mechanical properties of the EMWM were characterized by the density term, the strain strengthening term and the strain rate strengthening term in the Sherwood–Frost constitutive framework model. The advantages of the two constitutive models were fully combined to establish a relatively accurate quasi-static compression constitutive model of the plate-like EMWM with the consideration of the temperature effect. At the same time, the influence of thermal stress inside the EMWM should be considered.

Therefore, the new constitutive model for plate-like EMWM in wide temperature range can be initially expressed as Equation (7).

(7) $$\begin{array}{ll} \sigma & =\bar{H}\left(T\right)K(T)G\left(\rho \right)M\left(\varepsilon ,\dot{\varepsilon }\right)f\left(\varepsilon \right)+\sigma_{Thermal}\\ & =\left(1-T^{*m}\right)K(T)B(\frac{\rho }{\rho_{0}}{)}^{Y}M\left(\varepsilon ,\dot{\varepsilon }\right)f\left(\varepsilon \right)+\sigma_{Thermal} \end{array}$$

where *K*(*T*) is the thermal expansion correction coefficient.

The thermal stress of the EMWM changes with the change of the ambient temperature, and then the stress–strain curves will not start from zero strain and zero stress. To facilitate the analysis, thermal stress is only considered in temperature softening term.

### 3.1. Strain Rate Enhancement Term

**Figure 11:**
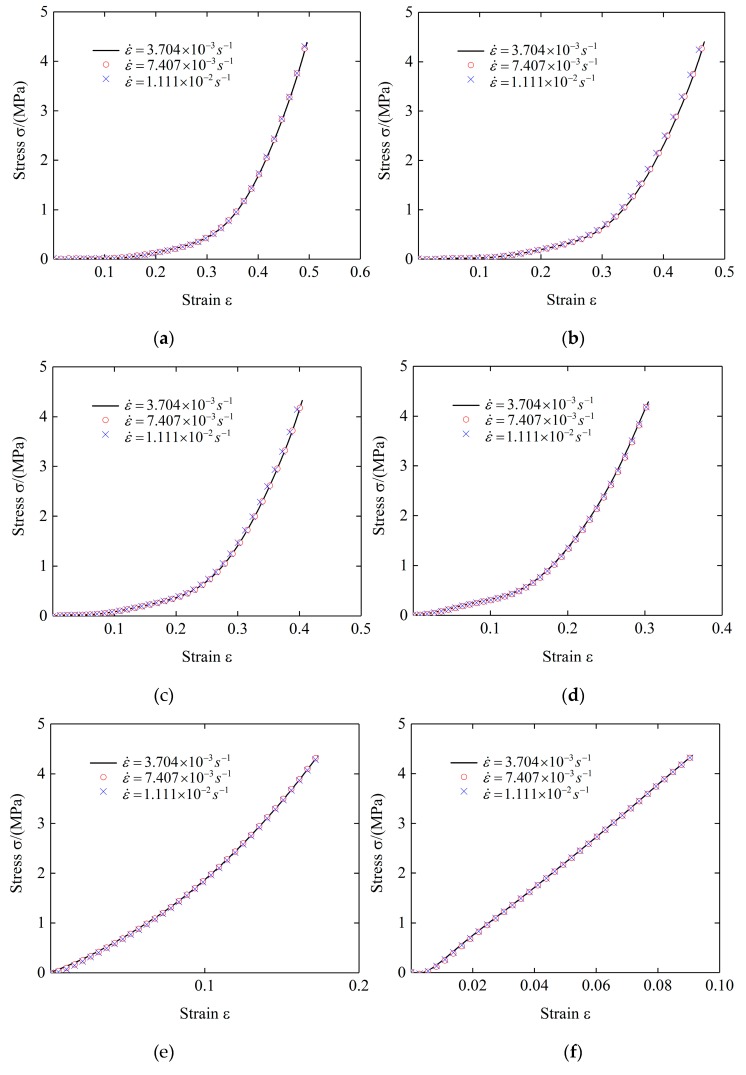
Stress–strain curves at different strain rates: (**a**) 20 °C; (**b**) 100 °C; (**c**) 200 °C; (**d**) 300 °C; (**e**) 400 °C; (**f**) 500 °C.

In this paper, the quasi-static performances of plate-like EMWM specimens were investigated in a wide temperature range (from room temperature to 500 °C). Under different temperature conditions, three small strain rates are taken for specimen 2, which are 3.704 × 10^−3^ s^−1^ (1 mm/min), 7.407 × 10^−3^ s^−1^ (2 mm/min), and 1.111 × 10^−2^ s^−1^ (1 mm/min). The test data is shown in Figure 11.

It can be seen from Figure 11 that in the temperature range from 20 °C to 500 °C, the changes of the strain rate have little effect on the stress–strain curve of the plate-like EMWM, and the influence of the strain rate can be ignored. This is due to that within this strain rate range, the slip speed of the wire helixes and the contact state of the internal contact point does not change significantly. Therefore, the strain rate strengthening term can be expressed as follows:

(8) $$M\left(\varepsilon ,\dot{\varepsilon }\right)=1$$

### 3.2. Shape Function

The elastic force of the plate-like EMWM can be described by the form of the product of the strain level and the density term, and can be expressed as follows:

(9) $$\sigma_{e}=G\left(\rho_{0}\right)\overset{n}{\underset{i=1}{\sum }}A_{i}\varepsilon^{i}$$

where *A_i_* is the coefficient of the polynomial, n is the number of polynomials.

The friction between the internal wire helixes at contact points of the EMWM directly affects the stiffness and the energy dissipation capacity of the EMWM. The internal contact points of the EMWM under the external load can be divided into three types: First type (1) there is no shearing force at the contact point. Second type (2) there is a shear force at the contact point, but no slip occurs due to the static friction at the contact point. Third type (3) there is a shear force at the contact point and the force is greater than the static friction force at the contact point. Slip friction occurs between the contact points.

The frictional contact point of the EMWM can be regarded as all of the contact points in the three-dimensional space, which satisfy the Poisson distribution characteristics. If the probability of the contact state in internal EMWM transitioning from the first type (1) to the second type (2) or the third type (3) is considered equal, and the mutual transition between states is completely independent, the latter can be considered a Poisson distribution. The relationship between the number $n_{3}$ of contact points where friction exists and the number of all contact points $n_{1}+n_{2}+n_{3}$ in the EMWM can be expressed by Equation (10).

(10) $$\frac{n_{3}}{n_{1}+n_{2}+n_{3}}=1-e^{-\lambda X}$$

where λ is the structural parameters of the EMWM, X is deformation value after alternating changes in deformation direction. $n_{1}$, $n_{2}$ and $n_{3}$ are respectively number of first type of contact points (1), second type of contact points (2) and third type of contact points (3), which directly affect the internal friction of EMWM components.

During the loading process of the plate-like EMWM, there exists a relative movement between the wire helixes, and the energy will be dissipated by friction. The stress on the EMWM includes the elastic stress $\sigma_{e}$ and the friction stress $\sigma_{f}$. The friction is related to the number of contact points and the contact area inside the EMWM. It can be known from the literature [28] that the relationship between the internal elastic stress $\sigma_{e}$ and the frictional stress $\sigma_{f}$ of the EMWM can be expressed as Equation (11).

(11) $$\sigma_{f}=\sigma_{e}\xi \left(1-e^{-\lambda \varepsilon }\right)$$

where *ξ* and *λ* are the proportional coefficients, which are associated with the contact points of the internal structure.

Then, the total stress inside the EMWM can be described as Equation (12).

(12) $$\sigma =\sigma_{f}+\sigma_{e}=\sigma_{e}\left[1+\xi \left(1-e^{-\lambda \varepsilon }\right)\right]$$

By comparing the Sherwood–Frost constitutive frame model with the above equation and substituting Equation (8) into Equation (2), the modified shape function can be obtained as Equation (13).

(13) $$\bar{f}\left(\varepsilon \right)=\left[1+\xi \left(1-e^{-\lambda \varepsilon }\right)\right]\overset{n}{\underset{i=1}{\sum }}A_{i}\varepsilon^{i}$$

The reference density $\rho_{0}$ is set as $1.905\;g/cm^{3}$, and the reference temperature *T*_0_ is set as 20 °C. When $T=T_{0}=T_{room}$, $\rho =\rho_{0}$, the temperature softening term and the density term can be expressed as Equations (14) and (15).

(14) $$H\left(T_{0}\right)=1-T^{*m}=1-\frac{\left(T_{0}-T_{room}\right)}{\left(T_{melt}-T_{room}\right)}=1$$

(15) $$G\left(\rho_{0}\right)=B(\frac{\rho_{0}}{\rho_{0}}{)}^{Y}=B$$

Substituting Equations (13)–(14) into the Equation (2), the stress–strain relationship of the plate-like EMWM specimen with a density of $1.905\;g/cm^{3}$ at 20 °C can be expressed as Equation (16).

(16) $$\begin{array}{ll} \sigma & =H\left(T_{0}\right)G\left(\rho_{0}\right)\bar{f}\left(\varepsilon \right)\\ & =B\left[1+\xi \left(1-e^{-\lambda \varepsilon }\right)\right]\sum_{i=1}^{n}A_{i}\varepsilon^{i}\\ & =\left[1+\xi \left(1-e^{-\lambda \varepsilon }\right)\right]\sum_{i=1}^{n}{A_{i}}^{\prime }\varepsilon^{i} \end{array}$$

where ${A_{i}}^{\prime }=B\cdot A_{i}$.

**Figure 12:**
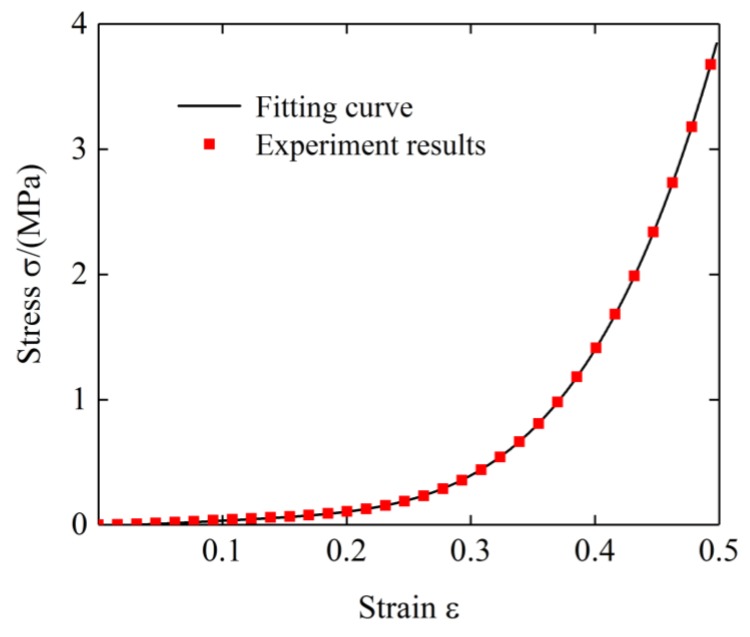
The stress-strain curves and its fitting curves ($\rho_{0}=1.905\;g/cm^{3}$, $T_{0}=20$ °C).

To obtain the correlation coefficient in the shape function, the test data of specimen 1 was subjected to the least squares fitting at room temperature. To avoid the shape function expression being too complicated and ensure high fitting precision, the value of *n* is set as 3. The fitting curve obtained is shown in Figure 12. The fitting parameters are shown in Table 2.

### 3.3. Density Term

**Figure 13:**
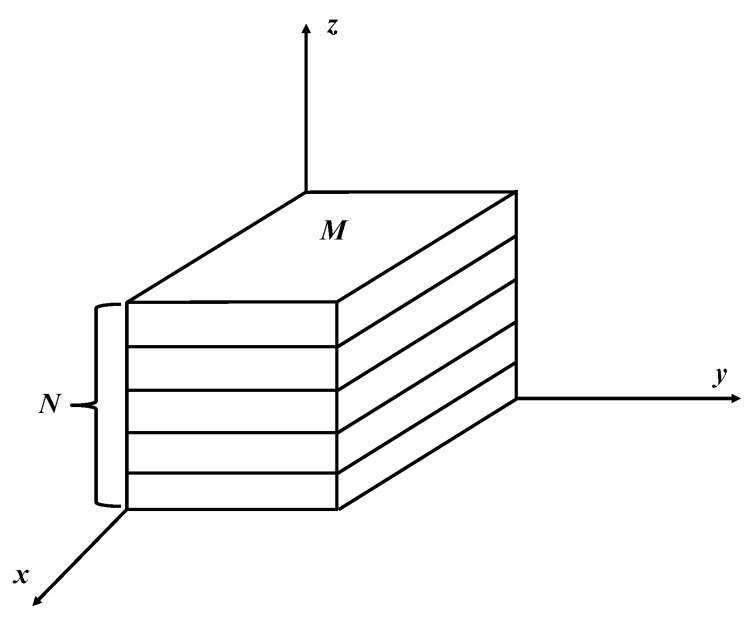
Division of the series-parallel structure.

The general expression of the density term has been given above, which is often used in the constitutive relationship of porous materials such as aluminum foam. The EMWM can be simplified to a series-parallel structure, as shown in Figure 13.

This volume unit has *M* structural units in its forming section, and there are *N* structural unit layers in the cross-section. For the unit volume of the plate-like EMWM, the following equation can be derived.

(17) $$MN=\frac{\rho_{MR}}{m_{u}}$$

where *m_u_* is the mass of the structural unit. 

Assuming that,

(18) $$\{\begin{array}{c} M=B_{1}\rho_{MR}{}^{X}\\ N=B_{2}\rho_{MR}{}^{1-X} \end{array}$$

where *B*_1_, *B*_2_ and *X* are the scale factor of the series-parallel structure.

**Figure 14:**
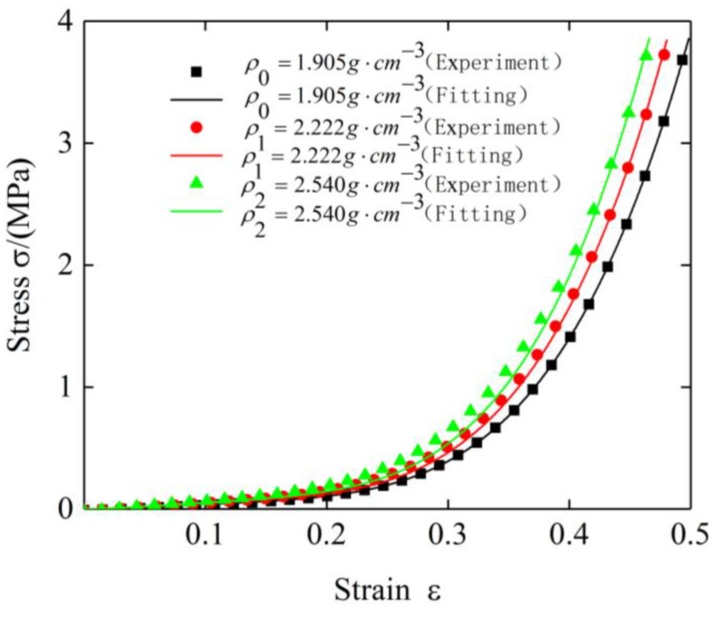
Stress–strain curves of plate-like EMWM with different densities and their fitting curves.

Considering the mechanical properties of the EMWM is similar to that of the aluminum foam, ignoring the coupling between strain and density, in this paper a way similar to the aluminum foam constitutive equation is used to express the effect of density on EMWM.

(19) $$G\left(\rho \right)=\frac{M}{N}=\frac{B_{1}\rho_{MR}{}^{X}}{B_{2}\rho_{MR}{}^{1-X}}=B(\frac{\rho }{\rho_{0}}{)}^{Y}$$

where *B* and *Y* are the reference coefficients of the density term. The static compression test data of the three specimens with different densities under room temperature were fitted, and the fitting results are shown in Figure 14. 

The values of *B* and *Y* were obtained by fitting to be 1 and 1.084, respectively. It can be seen from Figure 14 that the curve obtained by fitting fits the experimental data well, which indicates that the density term expression is feasible.

### 3.4. Temperature Softening Term

**Figure 15:**
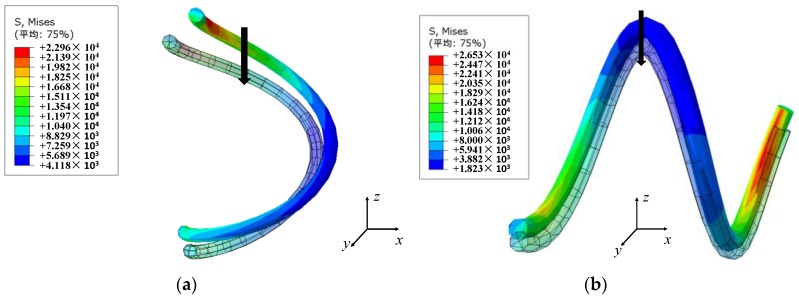
Expansion deformation of the wire helix in longitudinal and lateral arrangement: (**a**) Longitudinal; (**b**) Lateral.

To investigate the influence of the ambient temperature on the thermal expansion coefficient of the metal wire helixes, the thermal expansion behavior of the micro-element structure was analyzed by the finite element method (Abaqus). Assume that the inner wire helix of the plate-like EMWM has only longitudinal and lateral spiral arrangement [33], the material of the wire helix is 06Cr19Ni10, the Poisson’s ratio is 0.247, and the average expansion coefficient at 0–500 °C is $18.4\times 10^{-6}\;K^{-1}$, the average elastic modulus is $195\;kN/mm^{2}$, the diameter of the metal wire is 0.3 mm, the spring diameter of the wire helix is 3 mm, and the helix angle is 60°. A thermal load of 500 °C was applied to the wire helix, and the thermal expansion deformation of the longitudinal and lateral micro-body was compared with the wire helix at room temperature. The comparison results are shown in Figure 15. The z-axis in the Figure 15 indicates the forming direction and x-axis indicates the non-forming direction.

Figure 15 shows that the thermal deformation of the longitudinal micro-element is the axial direction deformation, and the thermal deformation of the lateral micro-element is the radial direction deformation. As shown in Figure 15, the micro-element has three-contact status under the molding pressure: no contact, slip contact and stick contact. Due to the existence of the internal porosity, the thermal expansion coefficient of the non-contact micro-element is smaller than the thermal expansion coefficient of the wire material itself, which is directly affected by the relative density of the EMWM. When thermal expansion occurs in the wire, the amount of radial expansion of the wire is much smaller than the amount of axial expansion of the wire. Thus, the $d_{s}$ can be considered as a constant. Assuming that before the temperature changes, the length of the wire in the $1\;cm^{2}$ EMWM can be expressed as Equation (20).

(20) $$l_{s}=\frac{4\rho_{MR}}{\pi \rho_{s}d_{s}{}^{2}}$$

where $\rho_{s}$ is wire density, $d_{s}$ is wire diameter.

The changes of wire density $\rho_{s}$ with temperature is ignored. When the amount of temperature change is ΔT and the linear expansion coefficient is $\alpha_{s}$, the changes in the length of the original length of $l_{s}$ is:

(21) $$\Delta l_{s}=\alpha_{s}l_{s}\Delta T=\alpha_{s}\frac{4\rho_{MR}}{\pi \rho_{s}d_{s}{}^{2}}\Delta T$$

At this time, the volume change of the wire is:

(22) $$\Delta V_{s}=\Delta l_{s}\frac{\pi d_{s}{}^{2}}{4}=\alpha_{s}\frac{\rho_{MR}}{\rho_{s}}\Delta T=\alpha_{s}\rho_{0}\Delta T$$

where $\rho_{0}=\frac{\rho_{MR}}{\rho_{s}}=\frac{V_{S}}{V_{MR}}$, which indicates the relative density of the EMWM. This reflects the proportional relationship between the volume of wire and pores in the EMWM. Changes in ambient temperature will cause changes in wire volume and cause changes of the contact state and the pores of the inner wire of the EMWM, with the result that the macroscopic performance of the EMWM will change.

The thermal expansion coefficient of the micro-elements in contact status is equivalent to the wire itself, and its elastic modulus is not affected by the parameters of the EMWM. With the increase of compression, the number of wire helixes in the non-contact status will decrease, and the internal porosity decreases. Thus, the influence of thermal expansion on the mechanical properties of plate-like EMWM is more significant with the increase of the amount of compression. According to the three contact types of the internal wire helixes (Figure 10), combined with the effect of thermal expansion on its contact type, the thermal expansion correction coefficient K(T) is assumed as follows:

(23) $$K(T)=C{\left(\frac{T}{T_{0}}-1\right)}^{z}e^{{\left(\frac{T}{T_{0}}+1\right)}^{p}}$$

where *C*, *z* and *p* are the proportional coefficients of the internal micro-element in non-contact, slip-contact and stick contact status, respectively.

There is a clear expression of the temperature softening term in the Johnson-Cook model, but it does not take into account the effects of thermal expansion of the material. In this paper, according to the trend of the stress-strain curve under different temperatures, an appropriate thermal expansion correction coefficient is added to the temperature softening term. The modified temperature softening term is expressed as follows

(24) $$\bar{H}\left(T\right)=\{\begin{array}{cc} H\left(T_{0}\right)=1, & T=T_{0}\\ \left(1-T^{*m}\right)\cdot K(T), & T>T_{0} \end{array}$$

When $T=T_{0}$, the EMWM is not affected by temperature softening term, and $\bar{H}\left(T_{0}\right)=H\left(T_{0}\right)=1$. The second term in the Equation (24) is the thermal expansion correction coefficient.

During the test, due to the pre-pressure (20 N), the EMWM was constrained by the upper head and lower support of the electronic universal testing machine in the molding direction. Therefore, in high-temperature environments, the wire helixes cannot expand freely in the molding direction. It indicates that as the temperature rises, the wire will expand internally and cause a decrease in the porosity of the EMWM. On the other hand, because EMWM was heated under restraint, the effect of thermal stress should be considered. The thermal stress values of the EMWM are shown in Table 3.

It is notable that at the same temperature, the greater the density of the specimens the greater the thermal stress. The reason for this is that the thermal conduction of EMWM is mainly divided into heat conduction of the wire and heat conduction of the internal air, wherein the wire conducts heat much faster than the internal air. On the one hand, the higher the density of the EMWM, the more the internal wire helixes in a given volume. It indicates that the EMWM has smaller porosity and is easier to conduct heat. On the other hand, the EMWM was constrained in the molding direction, the greater the density of the specimens, more wire helixes were heated and thermal expansion would occur, which would lead to a decrease in the porosity of metal rubber.

Under 500 °C, the maximum thermal stress accounts for 23.28% of the target stress, thus its impact cannot be ignored. Considering that the thermal stress is related to the ambient temperature and the density of the specimen, and its trend with temperature is close to the Gaussian fitting within 500 °C. Therefore, the density term is brought into the thermal stress calculation, assuming the coupling relationship of density and temperature is:

(25) $$\sigma (T,\rho )=\gamma e^{-(\frac{T-\tau }{\zeta }{)}^{2}}G\left(\rho \right)=\gamma e^{-(\frac{T-\tau }{\zeta }{)}^{2}}B(\frac{\rho }{\rho_{0}}{)}^{Y}$$

where γ, τ and ζ are the proportional coefficients of the thermal stress.

**Figure 16:**
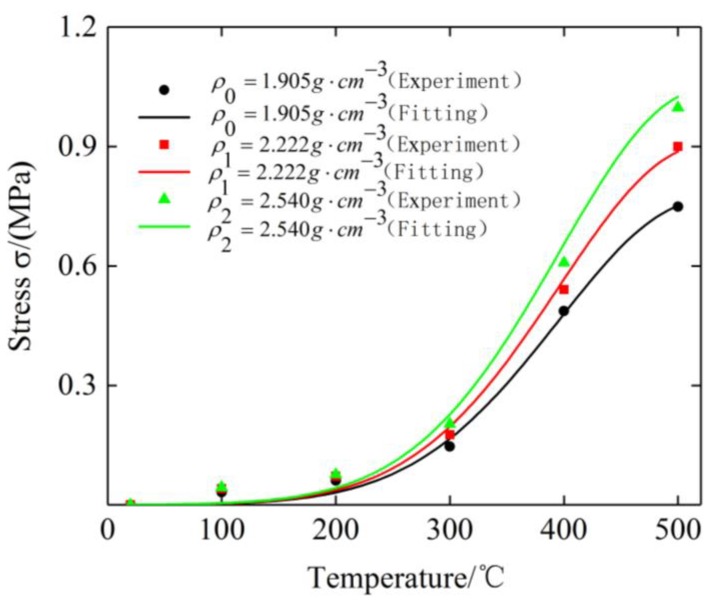
Stress–strain curves of three specimens at different temperatures and their fitting curves.

Figure 16 shows the fitting curves of three specimens under different ambient temperatures. The values of γ, τ and ζ obtained by fitting are 0.763, 523.3, and 180.9, respectively.

The total stress should be expressed as:

(26) $$\sigma_{Total}=\sigma +\sigma (T,\rho )$$

**Figure 17:**
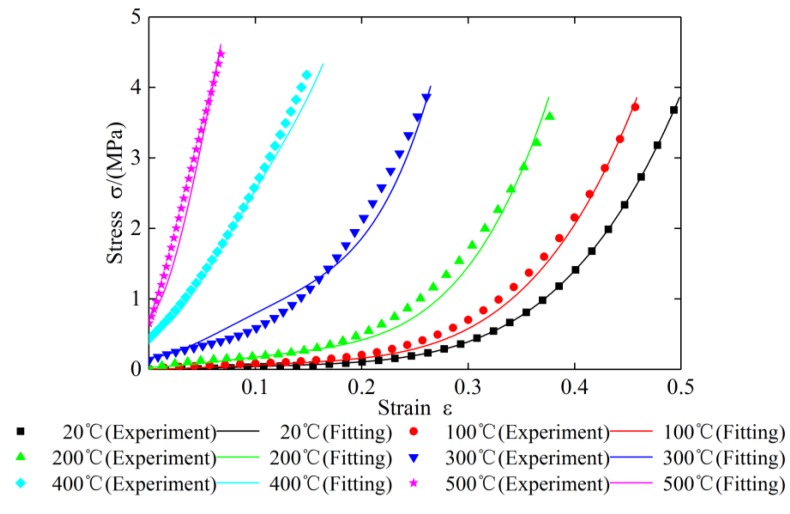
Stress–strain curves of specimen 1 at different temperatures and their fitting curves.

Bring the thermal stress data before being zeroed into the calculation. Figure 17 shows the fitting curves of specimen 1 ($1.905\;g/mm^{3}$) under different ambient temperatures. The values of C, *z* and *p* obtained by fitting are 0.1426, −0.8743, and 0.711161, respectively. A strain value was taken for every 100 test points of each curve, and the error between the test data and the fitted value is calculated. The fitting errors are shown in Table 4, Table 5, Table 6, Table 7 and Table 8.

It can be seen from Table 4, Table 5, Table 6, Table 7 and Table 8 that the error between the test value and the fitted value of the specimen 1 is less than 30% at 100 °C, the error between the test value and the fitted value of the specimen 1 is less than 20% at different temperatures except for 100 °C, indicating that the constitutive equation has relatively high reliability.

## 4. Constitutive Model Verification

The new constitutive model for plate-like EMWM in a certain density and temperature range can be obtained by the combination of Equations (8), (16), (19), (23) and (25). Therefore, the new constitutive model can be expressed by Equation (27).

(27) $$\begin{array}{l} \sigma =\left[1+\xi \left(1-e^{-\lambda \varepsilon }\right)\right]\overset{3}{\underset{i=1}{å}}({A_{I}}^{\prime }\varepsilon^{i})B(\frac{\rho }{\rho_{0}}{)}^{Y}\left(1-T^{*m}\right)g\left[C{\left(\frac{T}{T_{0}}-1\right)}^{z}e^{{\left(\frac{T}{T_{0}}+1\right)}^{p}}\right]\\ +\gamma e^{-(\frac{T-\tau }{\zeta }{)}^{2}}B(\frac{\rho }{\rho_{0}}{)}^{Y} \end{array}$$

where, $\rho_{0}$ is the reference density, and its value is 1.905 g/cm^3^; *T*_0_ is the reference temperature, and its value is 20 °C. The values of the other parameters of the constitutive model are shown in Table 9. 

This constitutive model is the combination of the Johnson–Cook model and the Sherwood–Frost constitutive framework. It includes the modified shape function $\bar{f}\left(\varepsilon \right)$, the density term G(ρ) which takes into account the effects of density on structural mechanical properties, the modified temperature softening term $\bar{H}\left(T\right)$, and the effect of thermal stress term $\sigma_{Thermal}$. The influence of the strain strengthening term is neglected in the new constitutive model.

**Figure 18:**
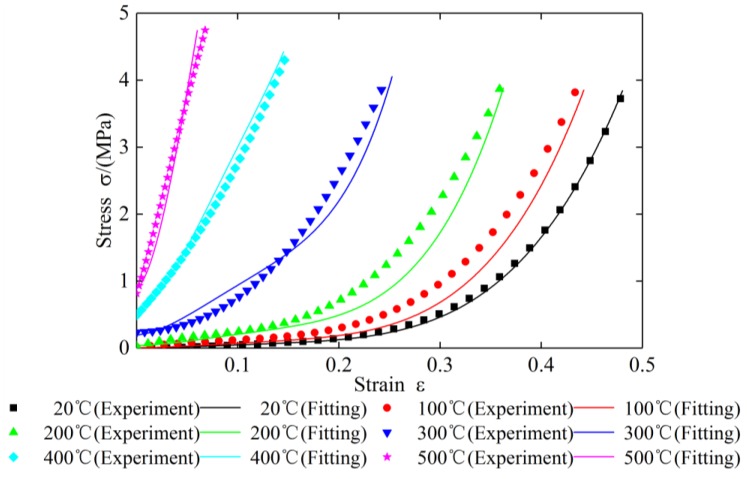
The comparison of stress–strain curves for specimen 2 under different temperatures.

**Figure 19:**
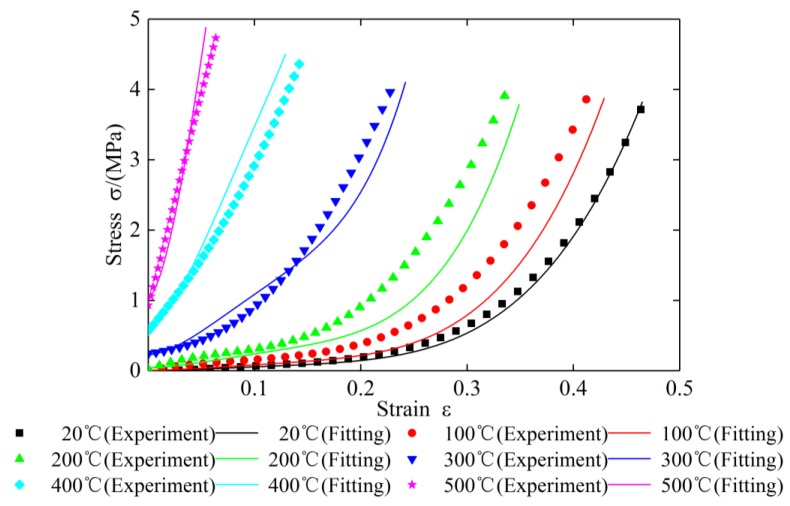
The comparison of stress–strain curves for specimen 3 under different temperatures.

To verify the correctness and feasibility of the new constitutive model, the test data of specimen 2 and specimen 3 under different temperatures are substituted into Equation (27) respectively. The comparison results of the theoretical curves with the experimental curves are shown in Figure 18 and Figure 19 and Table 10, Table 11, Table 12, Table 13, Table 14, Table 15, Table 16, Table 17, Table 18 and Table 19.

It can be seen from Figure 18 and Table 10, Table 11, Table 12, Table 13 and Table 14 that the calculated stress–strain values of specimen 2 under different temperatures matched well with curves drawn in accordance with the measured data except at 100 °C and 200 °C. At 100 °C and 200 °C, with the increase of compression, the test value will be greater than the theoretical value firstly, and then gradually tends to be the same. The maximum errors at 100 °C and 200 °C do not exceed 30%. It can be seen from Figure 19 and Table 15, Table 16, Table 17, Table 18 and Table 19 that comparison of the calculated values and measured data of specimen 3 under different temperatures are similar to those of specimen 2. The results of the comparison indicate that the accuracy of parameter identification was high, and the constitutive model for plate-like EMWM could properly describe the mechanical characteristics of the EMWM.

## 5. Conclusions

In this paper, a series of quasi-static compression tests for plate-like EMWM was carried out to investigate the effect of density and temperature on the mechanical properties of plate-like EMWM. A new constitutive model for plate-like EMWM was set up by the combination of the Johnson–Cook model and the Sherwood–Frost constitutive model. On the other hand, with the consideration of the contact status of the internal micro-element structure of the EMWM, the thermal expansion correction coefficient is added. The main conclusions, which can be drawn from the conducted experiments, are as follows:

(1) As the temperature increases, the amount of thermal expansion of the wire gradually increases, causing the internal gap of the EMWM to decrease drastically which makes the specimen enter the stiffened region earlier, and thus the non-linear characteristics of the EMWM will be weakened.

(2) In the wide temperature range (20–500 °C), the stiffness of the specimen gradually increase with the increase of the ambient temperature. At the same temperature, as the density of the specimen increases, the stiffness and the load-bearing capacity of the EMWM increases gradually.

(3) The physical meanings of the established constitutive model for plate-like EMWM are clear. The established constitutive model for EMWM can effectively predict the mechanical properties of plate-like EMWM.

## Figures and Tables

**Table 1:** Manufacture parameters for plate-like entangled metallic wire material (EMWM) specimens.

Number	Weight	Molding Pressure (kN/cm^2^)	Molding Density (g/cm^3^)
1	60 g	5.71	1.905
2	70 g	8.57	2.222
3	80 g	14.29	2.540

**Table 2:** Fitting parameters of shape function.

*n*	${A_{1}}^{\prime }$	${A_{2}}^{\prime }$	${A_{3}}^{\prime }$	ξ	λ
3	0.0586	−0.4145	1.111	326.4	0.4034

**Table 3:** The thermal stress values of the EMWM under different ambient temperature.

Density (g/cm^3^)	Thermal Stress (MPa)					
	20 °C	100 °C	200 °C	300 °C	400 °C	500 °C
1.905	0	0.03186	0.06086	0.14629	0.48686	0.749
2.222	0	0.041	0.07186	0.17586	0.54071	0.90014
2.540	0	0.04429	0.07629	0.20314	0.60829	0.99771

**Table 4:** The deviation between the tested stress and the fitted stress for specimen 1 under 100 °C.

ε	Test Value	Fitted Value	Error
0.04713	0.033327	0.023726	28.81%
0.09473	0.080497	0.057126	29.03%
0.14233	0.115817	0.091926	20.63%
0.18994	0.183317	0.142656	22.18%
0.23754	0.322787	0.248506	23.01%

**Table 5:** The deviation between the tested stress and the fitted stress for specimen 1 under 200 °C.

ε	Test Value	Fitted Value	Error
0.04008	0.090757	0.074125	18.33%
0.08057	0.161037	0.140075	13.02%
0.12105	0.213037	0.211575	0.69%
0.16153	0.316047	0.293335	7.19%
0.20202	0.517617	0.425835	17.73%

**Table 6:** The deviation between the tested stress and the fitted stress for specimen 1 under 300 °C.

ε	Test Value	Fitted Value	Error
0.02775	0.257606	0.265011	2.86%
0.05578	0.403966	0.453651	12.3%
0.0838	0.577176	0.669831	16.05%
0.11183	0.774946	0.887071	14.47%
0.13986	0.982516	1.113041	13.29%

**Table 7:** The deviation between the tested stress and the fitted stress for specimen 1 under 400 °C.

ε	Test Value	Fitted Value	Error
0.0163	0.660307	0.619834	6.13%
0.03276	0.990057	0.900274	9.07%
0.04922	1.327087	1.264424	4.72%
0.06568	1.702157	1.670464	1.86%
0.08214	2.116007	2.090454	1.21%

**Table 8:** The deviation between the tested stress and the fitted stress for specimen 1 under 500 °C.

ε	Test Value	Fitted Value	Error
0.00771	0.91779	0.891837	2.83%
0.01549	1.21027	1.154777	4.59%
0.02328	1.66119	1.517097	8.67%
0.03106	2.32797	1.957967	15.89%
0.03885	2.79517	2.460797	11.96%

**Table 9:** Parameters of the constitutive equation.

Parameter	${A_{1}}^{\prime }$	${A_{2}}^{\prime }$	${A_{3}}^{\prime }$	ξ	λ	*B*	*Y*	*m*	*C*	*z*	*P*	*γ*	*τ*	*ζ*
**Value**	0.0586	−0.4145	1.111	326.4	0.4034	1	1.084	1.196	0.1426	−0.8743	0.71116	0.763	523.3	180.9

**Table 10:** The deviation between the test and calculated values for specimen 2 under 100 °C.

ε	Test Value	Fitted Value	Error
0.0447	0.03708	0.026167	29.16%
0.08986	0.08484	0.063377	25.3%
0.07582	0.16602	0.122297	26.34%
0.18017	0.20987	0.152807	27.19%
0.22533	0.34775	0.251577	27.66%

**Table 11:** The deviation between the test and calculated values for specimen 2 under 200 °C.

ε	Test Value	Fitted Value	Error
0.037	0.102767	0.078557	23.56%
0.07437	0.203717	0.152477	25.15%
0.11174	0.271457	0.230357	15.14%
0.14911	0.389117	0.313407	19.46%
0.18648	0.600647	0.431177	28.21%

**Table 12:** The deviation between the test and calculated values for specimen 2 under 300 °C.

ε	Test Value	Fitted Value	Error
0.02577	0.272967	0.300246	10%
0.05179	0.422607	0.498656	18%
0.07782	0.634357	0.736136	16.04%
0.10384	0.797047	0.974686	22.29%
0.12987	1.139517	1.298445	13.95%

**Table 13:** The deviation between the test and calculated values for specimen 2 under 400 °C.

ε	Test Value	Fitted Value	Error
0.01608	0.740684	0.728833	1.6%
0.03231	1.081654	1.052863	2.66%
0.04855	1.425084	1.474813	3.49%
0.06479	1.804584	1.946523	7.87%
0.08103	2.222684	2.435563	9.58%

**Table 14:** The deviation between the test and calculated values for specimen 2 under 500 °C.

ε	Test Value	Fitted Value	Error
0.00771	1.086703	1.053698	3.04%
0.01549	1.406633	1.364218	3.02%
0.02328	1.918303	1.792128	6.58%
0.03106	2.589923	2.312798	10.7%
0.03885	3.062413	2.906638	5.09%

**Table 15:** The deviation between the test and calculated values for specimen 3 under 100 °C.

ε	Test Value	Fitted Value	Error
0.0425	0.038126	0.028345	25.65%
0.08543	0.095136	0.068975	27.5%
0.12837	0.154966	0.110895	28.44%
0.1713	0.222756	0.162405	27.10%
0.21423	0.358916	0.254505	29.09%

**Table 16:** The deviation between the test and calculated values for specimen 3 under 200 °C.

ε	Test Value	Fitted Value	Error
0.03457	0.115676	0.086025	25.63%
0.0695	0.225506	0.164555	27.03%
0.10442	0.319716	0.248765	22.19%
0.13935	0.443566	0.335395	24.39%
0.17427	0.624906	0.446255	28.59%

**Table 17:** The deviation between the test and calculated values for specimen 3 under 300 °C

ε	Test Value	Fitted Value	Error
0.02422	0.313243	0.335864	7.22%
0.04869	0.477863	0.548284	14.74%
0.07316	0.689843	0.801524	16.19%
0.09763	0.926873	1.061344	14.51%
0.1221	1.241003	1.321064	6.45%

**Table 18:** The deviation between the test and calculated values for specimen 3 under 400 °C.

ε	Test Value	Fitted Value	Error
0.01564	0.828886	0.834481	0.68%
0.03143	1.175986	1.193311	1.47%
0.04722	1.521106	1.662251	9.28%
0.06302	1.898686	2.189501	15.32%
0.07881	2.312106	2.738351	18.44%

**Table 19:** The deviation between the test and calculated values for specimen 3 under 500 °C

ε	Test Value	Fitted Value	Error
0.00749	1.224074	1.209984	1.15%
0.01505	1.618534	1.552764	4.06%
0.02261	2.182704	2.024564	7.25%
0.03018	2.749364	2.601074	5.39%
0.03774	3.212944	3.258314	1.41%
